# Patient-reported outcome measures for uncomplicated urinary tract infections in women: a systematic review

**DOI:** 10.1007/s11136-023-03358-5

**Published:** 2023-02-16

**Authors:** Katharina Piontek, Theresa Donhauser, Gesina Kann, Marie Fechtner, Christian Apfelbacher, Michaela Gabes

**Affiliations:** Institute of Social Medicine and Health Systems Research, Medical Faculty, Otto-von-Guericke University Magdeburg, Leipziger Str. 44, 39120 Magdeburg, Germany

**Keywords:** Uncomplicated urinary tract infections, Patient-reported outcomes measures, Questionnaire, Self-report, COSMIN

## Abstract

**Purpose:**

To conduct a systematic review of the quality of existing patient-reported outcome measures (PROMs) for use in women with uncomplicated urinary tract infections (UTIs) applying the COnsensus-based Standards for the selection of health Measurement INstruments (COSMIN) methodology, and to derive recommendations for their use in future research.

**Methods:**

A systematic literature search was performed in PubMed and Web of Science. Studies reporting on the development and/or validation of any PROMs for uncomplicated UTIs in women were considered eligible. We evaluated the methodological quality of each included study using the COSMIN Risk of Bias Checklist, and further applied predefined criteria for good measurement properties. Finally, we graded the evidence and derived recommendations for the use of the included PROMs.

**Results:**

Data from 23 studies reporting on six PROMs were included. From those, the Acute Cystitis Symptom Score (ACSS) and the Urinary Tract Infection-Symptom and Impairment Questionnaire (UTI-SIQ-8) can be recommended for further use. Both instruments showed sufficient content validity. We further found high-quality evidence for sufficient internal consistency of the UTI-SIQ-8, while this criterion was not assessed for the ACSS due to a formative measurement model. All other PROMs have the potential to be recommended for use, but require further validation.

**Conclusion:**

The ACSS and the UTI-SIQ-8 have the potential to be recommended for use in women with uncomplicated UTIs in future clinical trials. For all included PROMs, further validation studies are indicated.

Systematic review registration: PROSPERO.

**Supplementary Information:**

The online version contains supplementary material available at 10.1007/s11136-023-03358-5.

## Plain English summary

Uncomplicated urinary tract infections (UTIs) are among the most common bacterial infections in primary care typically affecting women. Several patient-reported outcome measures (PROMs) have been developed and validated to assess symptom burden, impairment of daily activities and health-related quality of life in women with uncomplicated UTIs. A systematic assessment of the quality of all existing PROMs using a standardized methodology has not been undertaken so far, but would enhance the selection of the most appropriate instrument. We aimed (a) to conduct a systematic review of the quality of all existing PROMs for women with uncomplicated UTIs using the COnsensus-based Standards for the selection of health Measurement INstruments (COSMIN) methodology, and (b) to derive recommendations for their use in future research. We included data from 23 studies reporting on six PROMs: the Acute Cystitis Symptom Score (ACSS), the Activity Impairment Assessment (AIA), the Urinary Tract Infection Symptom Assessment Questionnaire (UTISA), the International Consultation on Incontinence Questionnaire Female Lower Urinary Tract Symptoms (ICIQ-FLUTS), a symptom diary and the Urinary Tract Infection-Symptom and Impairment Questionnaire (UTI-SIQ-8). Our assessment revealed that the ACSS and the UTI-SIQ-8 can be recommended for use according to COSMIN criteria. Both instruments showed sufficient content validity. We further found high-quality evidence for sufficient internal consistency of the UTI-SIQ-8, while this criterion was not assessed for the ACSS due to a formative measurement model. All other PROMs have the potential to be recommended for use, but require further validation.

## Background

Urinary tract infections (UTIs) are among the most common bacterial infections in primary care and related to substantial individual and economic burden [1]. The classification of UTIs is based on their location in the urinary tract, symptoms, and complicating factors, and usually differentiates between uncomplicated and complicated UTIs [2]. Uncomplicated UTIs typically affect women, and with the exception of a spike in young women aged 14 to 24 years, the prevalence of UTIs increases with age [1, 3]. Evidence suggests that up to 60% of adult women will have at least one UTI in their life [4], and about 10% of postmenopausal women indicate that they had a UTI in the previous year [5]. Men are also at risk of developing UTIs. However, UTIs in men are considered complicated by definition [6]. Uncomplicated UTIs are generally self-limiting, but commonly treated with antibiotics as this therapy leads to a more rapid resolution of symptoms and is more likely to clear bacteriuria [3]. Though, this can result in long-term alteration of the normal microbiota of the vagina and the gastrointestinal tract, and in the development of multidrug-resistant microorganisms, which is a health threat itself of epidemic dimension [3]. For this reason, the identification and evaluation of new and effective strategies to prevent recurrences and alternative treatment strategies considering the patients' perspective are a high priority [7].

In medical research and care, patient-reported outcomes (PROs) are increasingly important as they provide unique information on health outcomes from the patient's perspective [8]. According to the guidelines of the US Food and Drug Administration (FDA), PROs are defined as “any report of the status of a patient’s health condition that comes directly from the patient, without interpretation of the patient’s response by a clinician or anyone else” [9]. Patient-reported outcome measures (PROMs) are standardized questionnaires for the assessment of PROs [10]. PROMs can be used to measure the impact of an intervention including disease symptoms or treatment side effects, functional outcomes such as physical, emotional and social functioning, or multidimensional constructs such as health-related quality of life (HRQoL) [11].

For UTIs, several disease-specific instruments assessing symptom burden [12–14], impairment of daily activities [12, 13], and HRQoL [14] have been developed and validated. Further, a variety of generic instruments has been used in studies on HRQoL of patients with UTIs including the short-from (SF)-36 and SF-12 questionnaires, the Health Utilities Index (HUI), the Quality of Well Being questionnaire (QWB), the Index of Well Being (IWB), and the Health and Activity Limitation index (HALex) [15]. Selecting a reliable and valid tool from the multitude of PROMs available is challenging. Systematic reviews of the quality of PROMs are helpful to inform instrument selection. According to guidance from the COnsensus-based Standards for the selection of health Measurement INstruments (COSMIN) initiative [16], systematic reviews should take both the quality of studies on measurement properties as well as the quality of the measurement properties themselves into account and then grade the whole body of evidence. A systematic assessment of the quality of all existing PROMs for use in women with uncomplicated UTIs and an evaluation of these PROMs using the COSMIN methodology has not been undertaken so far.

The aim of the present study was to conduct a systematic review of the quality of existing PROMs for use in women with uncomplicated UTIs and to derive recommendations for their use in future research.

## Methods

### Protocol and registration

This systematic review was conducted according to the recommendations of the Preferred Reporting Items for Systematic Reviews and Meta-Analyses Protocols (PRISMA-P) statement [17] and the COSMIN guideline and manual for systematic reviews of PROMs [16, 18]. The protocol has been registered in the International Prospective Register of Systematic Reviews (PROSPERO) (CRD42021290414).

### Literature search

A systematic literature search was conducted on 07 December 2021 using the databases PubMed and Web of Science. We used and adapted the search filter for finding studies on measurement properties of measurement instruments recommended by the COSMIN group [19]. The population and construct search was developed by the review team in collaboration with experts including a physician and a biologist working on uncomplicated UTIs. The search terms were compiled considering the ‘German Clinical Guideline on Epidemiology, Diagnostics, Therapy, Prevention, and Management of Uncomplicated Urinary Tract Infections in Adult Patients’ [20]. The search strategy included the following elements:

*A. Target population:* Women with uncomplicated UTIs including studies on recurrent UTIs. To allow for a broad sensitivity, we used a comprehensive compilation of controlled vocabulary and free text terms based on the literature.

*B. Construct of interest:* All PROMs related to uncomplicated UTIs.

*C. Measurement properties:* The validated and sensitive search filter for PubMed by Terwee et al. [19] was used and adapted for the search in Web of Science.

*D. Feasibility of PROMs:* The search strategy for this element was performed based on the search terms for the concept ‘feasibility’ of Heinl et al. (included in their search statement #1, Additional file 2) [21].

*E. Individual PROMs:* A list of PROMs in the context of uncomplicated UTIs already known was included.

*F. Exclusion filter:* We applied the filter by Terwee et al. [19] to exclude irrelevant publication types, animal studies and studies conducted in men.

The search syntax for PUBMED is displayed in Appendix 1. The single elements for search in PubMed were combined as follows: (((A AND B AND (C OR D)) OR (C AND E)) NOT F); in words: (((population AND construct AND (measurement properties OR feasibility)) OR (individual PROMs AND measurement properties)) NOT (exclusion filter)). For search in Web of Science, the search strategy for PubMed was adapted with appropriate syntax and index terms. There were no restrictions regarding publication date and language.

An update of our literature search capturing the publication date from December 07, 2021 to September 16, 2022 was conducted on September 16, 2022.

### Eligible studies

The eligibility criteria correspond to the COSMIN guideline for systematic reviews of PROMs [16]. Inclusion and exclusion criteria are depicted in Table 1. Eligible studies refer to any PROMs for women with uncomplicated UTIs, and at least 50% of the study sample needed to consist of women with uncomplicated UTIs. In case of unclear composition of the study population, we contacted the authors of the respective study to obtain detailed information. Further, the development of a PROM (“development paper”) and/or the evaluation of measurement properties (“validation paper”) needed to be the major aim of selected studies. Studies only using the PROM as an outcome measure and studies in which the PROM was used for the validation of another instrument were excluded. We included only full-text articles because abstracts often provide very limited information on the design of a study.

**Table 1:** Inclusion and exclusion criteria

	Inclusion criteria	Exclusion criteria
Population	Women with (recurrent) UTIs	Women with complicated UTIs; Women with other urological and gynecological lower reproductive tract infections
Study design	PROM development and/or validation study	All other study designs
Outcome	All patient-reported outcomes	Non-patient-reported outcomes, e.g., biomarkers, laboratory data
Type of measurement instrument	PROM	All others
Publication type	Articles with available full-text	Abstracts

*PROM* patient-reported outcome measure, *UTI* urinary tract infection

### Study selection

After deduplication of the records in Citavi 6, the screening of titles and abstracts was performed using Rayyan [22]. Two independent reviewers evaluated the titles and abstracts of the publications according to the inclusion and exclusion criteria to assess initial eligibility. We searched the full-texts for articles considered eligible at this stage, and these articles were also evaluated independently by two reviewers according to the predefined criteria. If any disagreement occurred, consensus was reached within the research team.

### Methodological assessment

Data on measurement properties were extracted from relevant studies in the following order:

- Evaluation of content validity.
- Evaluation of internal structure including structural validity, internal consistency, and cross-cultural validity/measurement invariance.
- Evaluation of the remaining measurement properties including reliability, measurement error, criterion validity, hypotheses testing for construct validity, and responsiveness.

All measurement properties were evaluated following three sub steps as outlined in the COSMIN manual (based on [16, 18, 23]). First, two reviewers independently evaluated the methodological quality of each single study on a measurement property using the COSMIN Risk of Bias checklist [18]. Both reviewers had a psychological background and were familiar with psychometrics and the COSMIN methodology. The COSMIN Risk of Bias checklist consists of 10 boxes containing all standards needed to assess the quality of a study on that specific measurement property (Table 2). Content validity is considered the most important measurement property because it is essential that all items of a PROM are relevant, comprehensive, and comprehensible regarding the construct of interest and the target population. PROM development is not considered a measurement property, but taken into account for the evaluation of content validity. Furthermore, the COSMIN group recommends that reviewers also give their own rating of the content of the PROM considering construct, target population, and context of use [23]. In the case that no content validity studies or only content validity studies of inadequate quality are available, and that the PROM development is of inadequate quality, the overall content validity rating is determined by the rating of the reviewers. The reviewers’ rating was performed according to our predefined criteria for the study population of interest as depicted in Table 1, and on the terms used for the definition of the target population and construct in the literature search (see Appendix 1). In case of uncertainty, the reviewers discussed the definition and relevance of a construct in view of the target population and the appropriateness of the items to reach consensus.

**Table 2:** Boxes of the COSMIN Risk of Bias checklist

Box 1	PROM development	Content validity
Box 2	Content validity	
Box 3	Structural validity	Internal structure
Box 4	Internal consistency	
Box 5	Cross-cultural validity/measurement invariance	
Box 6	Reliability	Remaining measurement properties
Box 7	Measurement error	
Box 8	Criterion validity	
Box 9	Hypotheses testing for construct validity	
Box 10	Responsiveness	

*COSMIN* Consensus-based Standards for the selection of health Measurement Instruments, *PROM* patient-reported outcome measure

Criterion validity refers to the degree to which the scores of a PROM are an adequate reflection of a gold standard. In the COSMIN group, consensus was reached that no gold standard exists for PROMs with the exception that a shortened instrument is compared with the original long version. In that case, the original long version is considered the gold standard, and it is recommended to consider the respective study as study on construct validity and to complete box 9 (hypotheses testing for construct validity). There were no validations of short vs long instrument versions in our systematic review. For the diagnosis of uncomplicated UTIs, urine analysis is considered the gold standard, with appropriate clinical examinations and typical symptom assessment [20]. However, such a diagnosis is not suitable to evaluate impact and bothersomeness of UTI or any PROs in UTI, but the clinical diagnosis lends itself for the evaluation of known-groups validity by comparing PROM scores of women with and without diagnosed UTI.

For interpreting the results of studies on hypotheses testing for construct validity, and on studies using a construct approach for the evaluation of responsiveness, a priori hypotheses were formulated for each PROM. For example, for the ACSS, we expected a positive correlation between the scores for typical symptoms and the values obtained from urine analyses, and further hypothesized that patients with UTI differ significantly from controls with respect to the scores in all domains of the ACSS. With respect to responsiveness, we expected improvement of the scores in all domains after antibiotic treatment. The evaluation of the quality of hypotheses testing for construct validity and responsiveness using a construct approach was performed according to the generic hypotheses as outlined in the COSMIN manual: (1) Correlations with (changes in) instruments measuring similar constructs should be ≥ 0.50, (2) Correlations with (changes in) instruments measuring related, but dissimilar constructs should be lower, i.e., 0.30–0.50, (3) Correlations with (changes in) instruments measuring unrelated constructs should be < 0.30, (4) Correlations defined under 1, 2, and 3 should differ by a minimum of 0.10; (5) Meaningful changes between relevant (sub)groups; and (4) AUC should be ≥ 0.70 for responsiveness.

The methodological quality of each study on a measurement property was rated on a 4-point rating scale as either very good, adequate, doubtful, or inadequate. The overall quality of a study was determined by the lowest rating of any standard in the box (“worst score counts”). Additionally, we extracted relevant data on characteristics of the included PROMs and study populations, and summarized these data in evidence tables. We further extracted data on interpretability and feasibility. These measurement properties are not formally evaluated by the COSMIN tools, but viewed as important considerations for the practical use of an outcome measure [24]. Interpretability refers to the ease of deriving meaning from an instrument's scores and includes the distribution of scores in the population, missing data, floor and ceiling effects, scores and change scores for relevant subgroups, minimal important change or difference, and information on response shift. Feasibility contains aspects of the ease of application, e.g., type and ease of administration, length, and completion time [18]. Second, the result of each single study on a measurement property was rated against the criteria for good measurement properties. Measurement properties were rated as either sufficient (+), insufficient (–), or indeterminate (?). Third, the quality of the evidence was summarized per measurement property per PROM. The summarized results were then rated against the criteria for good measurement properties (Table 3). The quality of evidence was graded using the Grading of Recommendations Assessment, Development, and Evaluation (GRADE) approach considering the methodological quality of studies, total sample size, and consistency of results [25]. In case of concerns regarding the trustworthiness of a result, the quality of evidence of the summarized results was downgraded per measurement property per PROM. Downgrading was possible due to risk of bias, inconsistency, imprecision, and/or indirectness. The quality of evidence was rated as either high, moderate, low, or very low. We did not grade the quality of evidence if an overall rating was indeterminate or inconsistent. Finally, to generate recommendations for the use of PROMs in future clinical trials, we categorized each PROM according to its methodological quality following the recommendations of the COSMIN group [16]:

A. PROMs with evidence for sufficient content validity (any level) and at least low-quality evidence for sufficient internal consistency.
B. PROMs categorized not in A or C.
C. PROMs with high-quality evidence for an insufficient measurement property.

**Table 3:** Criteria for good measurement properties

Measurement property	Rating	Criteria
Structural validity	+	**CTT** CFA: CFI or comparable measure > 0.95 OR RMSEA < 0.06 OR SRMR < 0.08^a^ **IRT/Rasch** No violation of unidimensionality^b^: CFI or TLI or comparable measure > 0.95 OR RMSEA < 0.06 OR SRMR < 0.08 *AND* No violation of local independence: residual correlations among the items after controlling for the dominant factor < 0.20 OR Q3’s < 0.37 *AND* No violation of monotonicity: adequate looking graphs OR item scalability > 0.30 *AND* Adequate model fit IRT: χ^2^ > 0.001 Rasch: infit and outfit mean squares ≥ 0.5 and ≤ 1.5 OR Z-standardized values > -2 and < 2
	?	CTT: not all information for ‘ + ’ reported IRT/Rasch: model fit not reported
	-	Criteria for ‘ + ’ not met
Internal consistency	+	At least low evidence^c^ for sufficient structural validity^d^” AND Cronbach’s alpha(s) ≥ 0.70 for each unidimensional scale or subscale^e^
	?	Criteria for “At least low evidence^c^ for sufficient structural validity^d^” not met
	-	At least low evidence^c^ for sufficient structural validity^d^ and Cronbach’s alpha(s) < 0.70 for each unidimensional scale or subscale^e^
Reliability	+	ICC or weighted Kappa ≥ 0.70
	?	ICC or weighted Kappa not reported
	-	ICC or weighted Kappa < 0.70
Measurement error	+	SDC or LoA < MIC^d^
	?	MIC not defined
	-	SDC or LoA > MIC
Hypotheses testing for construct validity	+	The result is in accordance with the hypothesis^f^
	?	No hypothesis defined (by the review team)
	-	The result is not in accordance with the hypothesis^f^
Cross-cultural validity/measurement invariance	+	No important differences found between group factors (such as age, gender, language) in multiple group factor analysis OR no important DIF for group factors (McFadden’s R^2^ < 0.02)
	?	No multiple group factor analysis OR DIF analysis performed
	-	Important differences between group factors OR DIF was found
Criterion validity	+	Correlation with gold standard ≥ 0.70 OR AUC ≥ 0.70
	?	Not all information for ‘ + ’ reported
	-	Correlation with gold standard < 0.70 OR AUC < 0.70
Responsiveness	+	The result is in accordance with the hypothesis^f^ OR AUC ≥ 0.70
	?	No hypothesis defined (by the review team)
	-	The result is not in accordance with the hypothesis^f^ OR AUC < 0.70

The criteria are based on Terwee et al. [19] and Prinsen et al. [16]

*AUC* area under the curve, *CFA* confirmatory factor analysis, *CFI* comparative fit index, *CTT* classical test theory, *DIF* differential item functioning, *ICC* intraclass correlation coefficient, *IRT* Item response theory, *LoA* limits of agreement, *MIC* minimal important change, *RMSEA* root mean square error of approximation, *SEM* standard error of measurement, *SDC* smallest detectable change, *SRMR* standardized root mean residuals, *TLI* Tucker–Lewis index.

“ + ” = sufficient, “- “ = insufficient, “?” = indeterminate

^a^To rate the quality of the summary score, the factor structure should be equal across studies

^b^Unidimensionality refers to a factor analysis per subscale, while structural validity refers to a factor analysis of a (multidimensional) patient-reported outcome measure

^c^As defined by grading the evidence according to the GRADE approach

^d^This evidence may come from different studies

^e^The criteria ‘Cronbach’s alpha < 0.95’ were deleted, as this is relevant in the development phase of a PROM and not when evaluating an existing PROM

^f^The results of all studies should be taken together and it should then be decided if 75% of the results are in accordance with the hypotheses

PROMs of category A can be recommended for use, and results obtained from these measures are considered trustworthy. PROMs of category B have the potential to be recommended for use, but require further validation. PROMs of category C should not be recommended for use. If only PROMs of category B are available, the PROM with the best evidence for content validity can be preliminarily recommended for use until further evidence is given [25].

## Results

### Literature search

**Fig. 1:**
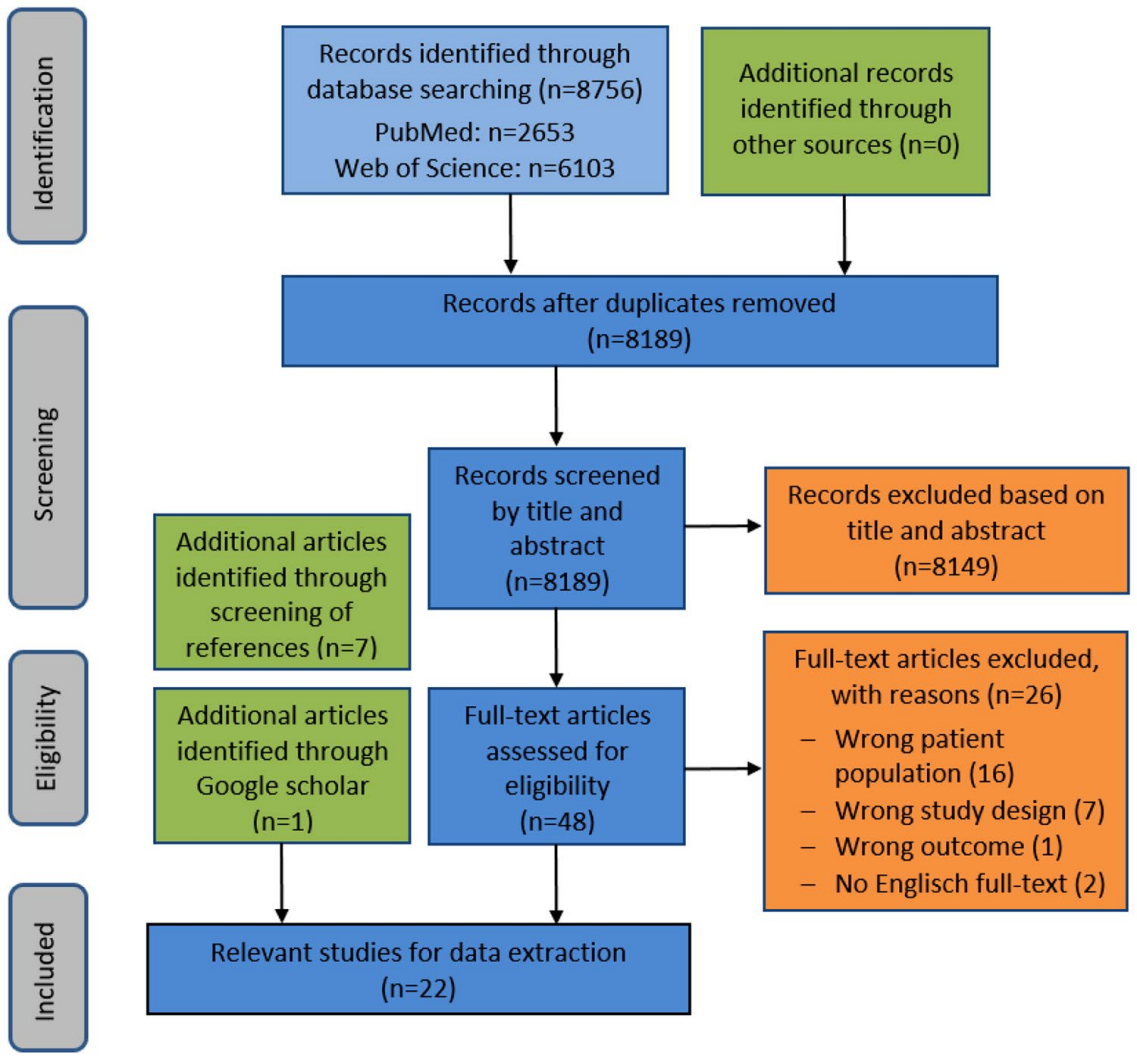
Adapted preferred reported items for systematic reviews and meta-analyses (PRISMA) protocols 2009 flow diagram

In total, our literature search yielded 8756 records (Fig. 1). After deduplication, 8189 records were screened, and 40 studies were considered eligible for full-text screening. We further identified seven relevant articles in the reference lists of the included studies and one article in Google Scholar, resulting in a total number of 48 studies for full-text screening. For data extraction, we included 22 studies reporting on six different PROMs. Fifteen studies reported on the Acute Cystitis Symptom Score (ACSS) [14, 26–39], and two studies, respectively, reported on the Activity Impairment Assessment (AIA) [40, 41] and on the Urinary Tract Infection Symptom Assessment Questionnaire (UTISA) [13, 42]. One study each was included reporting on the International Consultation on Incontinence Questionnaire Female Lower Urinary Tract Symptoms (ICIQ-FLUTS) [43], on a symptom diary [44], and on the Urinary Tract Infection-Symptom and Impairment Questionnaire (UTI-SIQ-8) [45].

The update of our literature search resulted in 564 records for title and abstract screening, of which two were considered eligible for full-text screening. Among those, one study reporting on the ACSS [46] fulfilled our inclusion criteria, and data were extracted.

### Characteristics of the included PROMs and study populations

An overview of the included PROMs and a description of the included study populations is given in Tables 4 and 5. All PROMs are self-administered and assess UTI symptoms and their severity, bothersomeness, and impact of UTI symptoms on daily activities and quality of life on a 4- or 5-point Likert scale. The ACSS additionally includes five questions on additional conditions which may affect therapy (menstruation, premenstrual syndrome, menopause, pregnancy, diagnosed diabetes mellitus), and five questions on the patient’s assessment of overall symptomatic changes after the baseline visit on a dichotomous scale (yes/no), respectively [27]. The sample sizes of the included studies ranged from 18 to 286 patients, and the overall age range was 15 to 87 years.

**Table 4:** Characteristics of the included PROMs

	ACSS	AIA	ICIQ-FLUTS	Symptom diary	UTISA	UTI-SIQ-8
Construct	Typical and differential symptoms and their severity, quality of life, and possible changes after therapy	Impact of UTI on work or other regular daily activities	Symptoms and bothersomeness	Symptom severity, bothersomeness and impact on daily activities	Symptoms and their severity, bothersomeness	Symptoms and their severity, bothersomeness
Target population	Women with AUC	Women with uncomplicated UTI	Women with LUTS	Adult patients with UTI	Women with uncomplicated UTI	Women with UTI
Mode of administration	Self-administered	Self-administered	Self-administered	Self-administered	Self-administered	Self-administered
Recall period	24 h	24 h	4 weeks	7 days	24 h	Respective day
(Sub)scales (number of items)	4 subscales (18 items): typical (6), differential (4), quality of life (3), additional (5)	0 subscales (5 items)	3 subscales (12 items, 2 questions each): filling symptoms (4), voiding symptoms (3), incontinence symptoms (5)	3 subscales (43 items): symptom severity (18), bothersomeness (18), impact on daily activities (7)	2 subscales (14 items): severity of symptoms (7 items), bothersomeness (7 items)	0 subscales (8 items: 4 items each to assess severity and bothersomeness)
Response options	4-point Likert scale: 0 (‘no symptom’) to 3 (‘severe’), 'Additional' subscale: yes/no-questions	5-point Likert scale: 0 (‘none of the time’) to 4 (‘all of the time’)	First question: 5-point Likert scale: 0 (‘none’) to 4 (‘four or more’) Second question: 11-point Likert scale: 0 (‘not at all’) to 10 (‘a great deal’)	4-point Likert scale: 0 (‘none’) to 3 (‘a lot’)	4-point Likert scale: 0 (‘did not have’) to 3 (‘severe’)	Symptom severity: 5-point Likert scale: 1 (‘not at all’) to 5 (‘very strong’) Bothersomeness: 5-point Likert scale 1 (‘not at all’) to 5 (‘very severe’)
Range of scores/scoring	Typical subscale: 0–18 Differential subscale: 0–12 Quality of life subscale: 0–9	Subscales 0-4; Overall score: 0 (no limitations) to 20 (maximum level of limitations)	Filling symptoms subscale: 0–16, Voiding symptoms subscale: 0–12, Incontinence symptoms subscale: 0–20	n/a	0–21	Symptoms: 1 (no symptoms at all) to 5 (very strong symptoms) Bothersomeness: 1 (no impairment at all) to 5 (very strong impairment)
Original language	Russian/Uzbek	English	English	Danish	English	German
Available translations	Chinese, French, German, Greek, Hungarian, Italian, Polish, Portuguese, Romanian, Russian, Spanish, Swedish, Tajik, Ukrainian, US/UK English, Uzbek	English, French	Chinese, Czech, Danish, Dutch, Finnish, French, German, Greek, Hungarian, Italian, Japanese, Norwegian, Persian, Polish, Portuguese, Spanish, Swedish, Tamil, Thai, Turkish, US/UK English	Danish, English	13 languages (no detailed data available)	English, German

*AUC* acute uncomplicated cystitis, *LUTS* lower urinary tract symptoms*, PROM* patient-reported outcome measure*, UTI* urinary tract infection

*ACSS* Acute Cystitis Symptom Score, *AIA* Activity Impairment Assessment Score, *ICIQ-FLUTS* International Consultation on Incontinence Questionnaire Female Lower Urinary Tract Symptoms, *UTISA* Urinary Tract Infection Symptom Assessment Questionnaire, *UTI-SIQ-8* Urinary Tract Infection-Symptom and Impairment Questionnaire

**Table 5:** Characteristics of the included study populations

Instrument	Reference	Sample size	Age mean (*SD*) or median	Setting	Country (Language)	Measurement properties
ACSS	Alidjanov et al. [14]	286	*M* = 32.3 (± 12.3), range 15–73 years	Outpatient clinic	Uzbekistan (Uzbek), Russia (Russian)	PROM development, internal consistency, construct validity, responsiveness
	Alidjanov et al. [26]	83	*M* = 35.6 (± 13.7), range 19–83 years	Centre of Urology	Uzbekistan (Uzbek), Russia (Russian)	Content validity, internal consistency, construct validity, responsiveness
	Alidjanov et al. [27]	36	Median = 40 (IQR = 21–59), range 18–90 years	Urological clinic	Germany (German)	Content validity, internal consistency, construct validity
	Alidjanov et al. [28]	63	*M* = 30.7 (± 11.5), range 17–64 years	Outpatient clinic	Uzbekistan (Uzbek), Russia (Russian)	Internal consistency, responsiveness
	Alidjanov et al. [29]	18	*M* = 48.4 (± 21.6), range 16–80 years	Urological practice	United Kingdom (English)	Content validity, internal consistency, construct validity
	Alidjanov et al. [30]	228 (follow-up: 48)	*M* = 31.49 (± 11.71)	Outpatient clinic	Uzbekistan (Uzbek), Russia (Russian)	Internal consistency, construct validity, responsiveness
	Alidjanov et al. [31]	48	*M* = 31.10 (± 10.64), range 19–63 years	Outpatient clinic	Uzbekistan (Uzbek), Russia (Russian)	Internal consistency, responsiveness
	Alidjanov et al. [32]	134	*M* = 36.28 (± 16.03), range 17–82 years	Data extraction: e-USQOLAT	German, Hungarian, Tajik, Uzbek	Responsiveness
	Alidjanov et al. [33]	167	*M* = 36.8 (± 15.3), range 17–87 years	Clinical site	United States (English)	Internal consistency, construct validity, responsiveness
	Alidjanov et al. [34]	97	Median = 41 (IQR = 28–57), range 17–83 years	Physician’s office	Switzerland (German)	Internal consistency, construct validity, responsiveness
	Bruyère et al. [35]	17	Median = 46, range 20–85 years	NR	France (French)	Content validity
	Choi et al. [46]	50 (patients: 31, controls: 19)	M = 54.4 (± 15.5)	Clinical setting	Korea (Korean)	Content validity, internal consistency, construct validity
	Di Vico et al. [36]	100 (patients: 54, controls: 46)	Patients: median = 36 (IQ R = 28–49) Controls: median = 38 (IQR = 29–45)	Outpatient office and fertility center	Italy (Italian)	Construct validity
	Magyar et al. [37]	68 (patients: 31, controls: 37)	Patients: median = 42, range 18–78 years Controls: median = 48, range 19–85 years	Urological outpatient clinic	Hungary (Hungarian)	Content validity, internal consistency, construct validity, responsiveness
	Stamatiou et al. [38]	30	*M* = 23.84, range 22–89 years	NR	Greece (Greek)	Content validity
	Stamatiou et al. [39]	92	*M* = 46.7 (± 16.6)	Clinical setting	Greece (Greek)	Internal consistency, construct validity, responsiveness
**AIA**	Wild et al. [40]	276	*M* = 33.0 (± 11.46), range 18–78 years	Clinical setting	United States (English)	Structural validity, internal consistency, construct validity, responsiveness
	Vincent et al. [41]	50	*M* = 44.6, range 20–52	Clinical setting	France (French)	Content validity, structural validity, internal consistency, construct validity, responsiveness
**ICIQ-FLUTS**	Chattrakulchai et al. [43]	283 (clinical population: 142, community population: 141)	Clinical population: *M* = 65.08 (± 11.68) Community population: *M* = 55.25 (± 12.76)	Urogynecology clinic	Thailand (Thai)	Content validity, internal consistency, reliability, construct validity
**Symptom diary**	Holm et al. [44]	451	NR	Practitioner’s office	Denmark (Danish)	PROM development, content validity, structural validity, internal consistency, cross-cultural validity/measurement invariance
**UTISA**	Chang et al. [42]	293 (patients: 169, controls: 124)	Patients: *M* = 44.0 (± 13.7) Controls: *M* = 44.6 (± 12.3)	Clinical setting	Taiwan (Chinese)	Structural validity, internal consistency, construct validity, responsiveness
	Clayson et al. [13]	276	*M* = 33.0 (± 11.46), range 18–78 years	Electronic format	United States (English)	Structural validity, internal consistency, construct validity, responsiveness
**UTI-SIQ-8**	Gágyor et al. [45]	120	*M* = 43.3 (± 16.6)	Primary care practices	Germany (German)	PROM development, structural validity, internal consistency, reliability, construct validity, responsiveness

*IQR* interquartile range*, NR* not reported, *PROM* patient-reported outcome measure, *SD* standard deviation

*ACSS* Acute Cystitis Symptom Score, *AIA* Activity Impairment Assessment Score*, ICIQ-FLUTS* International Consultation on Incontinence Questionnaire Female Lower Urinary Tract Symptoms, *UTISA* Urinary Tract Infection Symptom Assessment Questionnaire, *UTI-SIQ-8* Urinary Tract Infection-Symptom and Impairment Questionnaire

### Information on interpretability and feasibility

No data regarding interpretability and feasibility were reported for the ICIQ-FLUTS [43] and the symptom diary [44]. Information on the distribution of scores in the study population were given for the ACSS, the AIA, and the UTI-SIQ-8. One study reporting on the preliminary clinical validation of the UK English version of the ACSS showed that almost all variables were distributed with a skewness and kurtosis close to zero among patients and controls [29]. For the ACSS, a total score of six points in the domain of typical symptoms was established to predict acute cystitis [30]. It has been further demonstrated that success/cure and non-success/failure of therapy can be clearly differentiated by the scores obtained in the ‘typical symptoms’ and ‘quality of life’ domains [31]. Regarding the AIA, both included studies reported a non-normal distribution of the total score [40, 41]. The study evaluating the French adaptation of the AIA [41] additionally found that the distribution of answers to each item on day 0 showed a slight floor effect on the first item (‘cut down on amount of time spent at work or other activities’). The items of the UTI-SIQ-8 [45], which was completed at baseline and for seven days consecutively, showed a low level of skewness at baseline, and the distribution was more positively skewed on the following days. Data on minimal important difference (MID) were reported for the UTISA [13], which is completed at baseline and at 3-h and 8-h intervals until all symptoms are resolved. Analyses showed that the MID was 1.75 for urination regularity, 1.50 for problems with urination, 1.25 for pain associated with UTI, and 0.50 for blood in urine. For all included PROMs, no data were available regarding missing data, scores, and change scores for relevant subgroups and response shift.

With respect to feasibility, no study has reported difficulties regarding the patient’s comprehensibility and administration of the PROM. The study on the UTI-SIQ-8 used an online app and reported that this mode of administration was the women’s preferred form to complete the questionnaire over the paper-and-pencil version [45]. One study on the UTISA also used an electronic format for data collection [13]. The ACSS, the AIA, and the UTISA are copyrighted. For all instruments, it was stated that they are easy to use and completed in a short time by patients with applicability in both research and clinical practice. Information on access to all identified PROMs is given in Appendix 2.

### Measurement properties of instruments

When extracting the data using the COSMIN Risk of Bias checklist, we assessed the agreement of the reviewer for each box per study and calculated the overall agreement. The reviewers had a mean agreement of 76.5% across all studies. Major disagreements were discussed with a third reviewer having expertise with the COSMIN methodology.

#### Evaluation of content validity

The results of the overall content validity rating are displayed in Appendix 3. The PROM development studies for the AIA [40], the ICIQ-FLUTS [47] and the UTISA [12] were rated ‘inadequate’ since a sample from the target population was not involved in the development of the PROM. The PROM development studies of the ACSS [14] and the symptom diary [44] were rated ‘doubtful’ due to methodological weaknesses regarding the collection and analysis of qualitative data for PROM design, and due to methodological weaknesses of the pilot test. The UTI-SIQ-8 received an ‘inadequate’ PROM development rating because the development process did not include a sample representing the target population, and a cognitive interview study or other pilot test was not conducted [45]. The content validity studies of the ACSS [14, 27, 29, 35, 37, 38, 46], the AIA [41], the ICIQ-FLUTS [43], and the symptom diary [44] were rated ‘doubtful’ because detailed information about different aspects of the procedure were not provided. No content validity studies were performed for the UTISA and the UTI-SIQ-8.

The quality of evidence was rated ‘moderate’ for the ACSS and the symptom diary because at least one content validity study of doubtful quality was available (Appendix 4). For the UTI-SIQ-8, the quality of evidence was rated ‘very low’ because only a PROM development study of inadequate quality was conducted, and the evaluation of the content validity was based solely on the reviewers’ rating. Due to the inadequate PROM development study and the lack of a content validity study, the content validity assessment of the UTISA was based on the reviewers’ rating, and the quality of evidence was therefore rated ‘very low.’

As we found no high-quality evidence for insufficient content validity of any PROM, we subsequently assessed the remaining measurement properties of each PROM.

#### Evaluation of the remaining measurement properties

We assessed structural validity, internal consistency, cross-cultural validity/measurement invariance, reliability, hypotheses testing for construct validity and responsiveness. The results of the evaluation of the quality of studies on measurement properties and the rating of the methodological quality of the instruments are displayed in Table 6. In total, the methodological quality of 63 studies on measurement properties was evaluated. Among those, 26 (41.3%) had very good, 17 (27.0%) had adequate, 14 (22.2%) had doubtful, and 6 (9.5%) had inadequate methodological quality. No study has analyzed measurement error.

**Table 6:** Quality of studies on measurement properties and methodological rating of the instruments

PROM	Reference	Methodological quality (rating^a^)					
		Structural validity^b^	Internal consistency^b^	Cross-cultural validity/ measurement invariance	Reliability	Hypotheses testing	Responsiveness
ACSS	Alidjanov et al. [14]					Very good (+), Adequate (+)	Doubtful (+)
	Alidjanov et al. [26]					Very good (+/+), Doubtful (+)	Inadequate (?)
	Alidjanov et al. [27]					Very good (+/+)	
	Alidjanov et al. [28]						Doubtful (+)
	Alidjanov et al. [29]					Adequate (+)	
	Alidjanov et al. [30]					Doubtful (+)	
	Alidjanov et al. [31]						Very good (+), Doubtful (+)
	Alidjanov et al. [32]						Doubtful (?)
	Alidjanov et al. [33]					Very good (+), Doubtful (–)	Very good (+)
	Alidjanov et al. [34]					Very good (–), Adequate (–)	Very good (+)
	Bruyère et al. [35]						
	Choi et al. [46]					Very good (+), Doubtful(+)	
	Di Vico et al. [36]					Very good (+)	
	Magyar et al. [37]						
	Stamatiou et al. [38]						
	Stamatiou et al. [39]					Very good (+), Adequate (–)	Adequate (?)
AIA	Wild et al. [40]	Very good (?), Adequate (?)	Very good (?)			Very good (+), Adequate (+)	Very good (+)
	Vincent et al. [41]	Adequate (?)	Doubtful (?)			Adequate (+), Doubtful (–)	Very good (–)
ICIQ-FLUTS	Chattrakulchai et al. [43]		Doubtful (?)		Inadequate (?)	Very good (+)	
Symptom diary	Holm et al. [44]	Very good (?)	Very good (?)	Inadequate (+)			
UTISA	Chang et al. [42]	Adequate (?)	Doubtful (?)			Very good (+)	Adequate (+), Doubtful (+), Inadequate (?)
	Clayson et al. [13]	Adequate (?)	Very good (?)			Very good (+), Adequate (+/+)	Inadequate (+/+)
UTI-SIQ-8	Gágyor et al. [45]	Very good (+), Adequate (?)	Very good (+)		Doubtful (–)	Adequate (+)	Adequate (+)

*ACSS* Acute Cystitis Symptom Score, *AIA* Activity Impairment Assessment Score, *ICIQ-FLUTS* International Consultation on Incontinence Questionnaire Female Lower Urinary Tract Symptoms, *UTISA* Urinary Tract Infection Symptom Assessment Questionnaire, *UTI-SIQ-8* Urinary Tract Infection-Symptom and Impairment Questionnaire

^a^(+) sufficient rating, (–) insufficient rating, (?) indeterminate rating

^b^Not applicable for the ACSS due to a formative measurement model.

Measurement error was not investigated in the included studies

#### Summary of the findings and grading of the quality of evidence

The summarized results per measurement property and PROM are depicted in Table 7. We did not evaluate the structural validity and internal consistency of the ACSS due to a formative measurement model.

**Table 7:** Summary of findings

PROM	Summary or pooled result	Overall rating	Quality of evidence
*ACSS*			
Internal consistency	Data not reported due to the formative measurement model	Quality not evaluated due to the formative measurement model	
Hypotheses testing	13 out of 17 hypotheses confirmed, sample size: 1461	Sufficient	Moderate (due to inconsistency)
Responsiveness	7 out of 7 hypotheses confirmed, sample size: 684	Sufficient	High
*AIA*			
Structural validity	Not all information for sufficient rating reported, sample size: 326	Indeterminate	–
Internal consistency	Alpha = 0.93, no evidence for sufficient structural validity, sample size: 326	Indeterminate	–
Hypotheses testing	1 out of 2 hypotheses confirmed, sample size: 326	Inconsistent	High
Responsiveness	1 out of 1 hypothesis confirmed, sample size: 326	Sufficient	High
*ICIQ-FLUTS*			
Internal consistency	Alpha = 0.65–0.85, no evidence for sufficient structural validity, sample size: 283	Indeterminate	–
Reliability	Not all information for sufficient rating reported, sample size: 283	Indeterminate	–
Hypotheses testing	1 out of 1 hypothesis confirmed, sample size: 283	Sufficient	High
*Symptom diary*			
Structural validity	Not all information for sufficient rating reported, sample size: 173–376	Indeterminate	–
Internal consistency	Alpha = 0.55–0.94, no evidence for sufficient structural validity, sample size: 173–376	Indeterminate	–
Cross-cultural validity/Measurement Invariance	No important differential item functioning, sample size: 173–376	Sufficient	Very low (due to risk of bias)
*UTISA*			
Structural validity	Not all information for sufficient rating reported, sample size: 445	Indeterminate	–
Internal consistency	Alpha = 0.72–0.87, no evidence for sufficient structural validity, sample size: 445	Indeterminate	–
Hypotheses testing	4 out of 4 hypotheses confirmed, sample size: 569	Sufficient	High
Responsiveness	4 out of 4 hypotheses confirmed, sample size: 338	Sufficient	10b. Low (due to risk of bias), 10c. Moderate (due to risk of bias), 10d. Low (due to risk of bias)
*UTI-SIQ-8*			
Structural validity	SRMR = 0.066, sample size: 120	Sufficient	High
Internal consistency	Alpha = 0.86–0.91, sample size: 120	Sufficient	High
Reliability	ICC = 0.32, sample size: 120	Insufficient	Low (due to risk of bias)
Hypotheses testing	1 out of 1 hypothesis confirmed, sample size: 120	Sufficient	Moderate (due to risk of bias)
Responsiveness	1 out of 1 hypothesis confirmed, sample size: 120	Sufficient	Moderate (due to risk of bias)

Not applicable to the ACCS due to a  formative measurement model

*ICC* intraclass correlation coefficient*, PROM* patient-reported outcome measure, *SRMR* standardized root mean squared residual

*ACSS* Acute Cystitis Symptom Score, *AIA* Activity Impairment Assessment Score, *ICIQ-FLUTS* International Consultation on Incontinence Questionnaire Female Lower Urinary Tract Symptoms, *UTISA* Urinary Tract Infection Symptom Assessment Questionnaire, *UTI-SIQ-8* Urinary Tract Infection-Symptom and Impairment Questionnaire

#### Recommendation

According to COSMIN criteria, the ACSS and the UTI-SIQ-8 were placed into category A, and all other PROMs were placed into category B (Table 8).

**Table 8:** Recommendations for use in future clinical trials

PROM	Category A		Category C	Recommendation
	Sufficient content validity (any level)	At least low-quality evidence for sufficient internal consistency	High-quality evidence for an insufficient measurement property	
ACSS	✓	Not applicable	×	A
AIA	×	×	×	B
ICIQ-FLUTS	×	×	×	B
Symptom diary	✓	×	×	B
UTISA	×	×	×	B
UTI-SIQ-8	✓	✓	×	A

*PROM* patient-reported outcome measure

*ACSS* Acute Cystitis Symptom Score, *AIA* Activity Impairment Assessment, *ICIQ-FLUTS* International Consultation on Incontinence Questionnaire Female Lower Urinary Tract Symptoms, *UTISA* Urinary Tract Infection Symptom Assessment Questionnaire, *UTI-SIQ-8* Urinary Tract Infection-Symptom and Impairment Questionnaire

## Discussion

The present systematic review is the first to provide a synthesized methodological evaluation of the measurement properties of PROMs for use in women with uncomplicated UTIs applying the COSMIN methodology. We extracted data from 23 studies reporting on six PROMs. Our assessment revealed that the ACSS and the UTI-SIQ-8 can be recommended for use in future clinical trials (COSMIN category A). We further found that the AIA, the ICIQ-FLUTS, the symptom diary, and the UTISA have the potential to be recommended for use, but need further validation (COSMIN category B). Although the ACSS and the UTI-SIQ-8 met the requirements for a recommendation, some evidence gaps remain. All included PROMs have substantial conceptual and methodological weaknesses, which need to be considered.

The classification of a PROM into a recommendation category according to COSMIN is based on the evaluation of content validity and structural validity. With respect to the assessment of content validity, the involvement of patients is significant as all items of a PROM should be relevant for the construct of interest (within a specific population and context of use), comprehensive with respect to patient concerns, and understood by patients as intended [23]. Furthermore, it is important for PROM development to involve affected patients via interviews or focus groups in the item generation phase since this is often required by regulatory authorities [48]. The development of the ACSS was based on the Urinary Symptoms and Quality of Life Assessment Tool (USQOLAT) [49], and it is unclear whether patients were asked about comprehensibility and comprehensiveness of the items in the pilot studies. For the ACSS, seven content validity studies were available [14, 27, 29, 35, 37, 38, 46]. In these studies, patients were asked about comprehensibility, but not about relevance and comprehensiveness. The development of the UTI-SIQ-8 involved a multiprofessional team of healthcare professionals, but not patients. A content validity study was not performed. Thus, our evaluation was based only on the reviewers’ rating. Regarding the PROMs in category B, the symptom diary fulfilled the criteria for sufficient content validity. The development of the symptom diary included the assessment of comprehensibility and comprehensiveness from the patient’s perspective, and relevance, comprehensiveness and comprehensibility were assessed from the patient’s perspective in a content validity study.

Structural validity refers to the degree to which the scores of an instrument are an adequate reflection of the dimensionality of the construct to be measured. We found sufficient structural validity only for the UTI-SIQ-8. The evaluation of the internal structure including structural validity is relevant for PROMs that are based on a reflective model, in which a construct manifests itself in the items, i.e., the items are a reflection of the construct to be measured. The ACSS is based on a formative measurement model, and therefore structural validity was not assessed. The ACSS is hypothesized to measure typical and differential symptoms, quality of life and possible changes after therapy, but the underlying measurement model has not been analyzed yet.

Although the focus of this review was on the evaluation of the psychometric properties of the identified PROMs, the intended use is crucial when selecting a PROM. The PROMs included in the present study focus on different domains, which is of importance for their application in clinical care and research. The ACSS includes the assessment of symptom severity, the patient’s quality of life, the differentiation from other urological and gynecological diseases, and the assessment of conditions which may affect therapy. The AIA is measuring the impact of UTI on work and other regular daily activities. The purpose of the ICIQ-FLUTS, the UTISA, and the UTI-SIQ-8 is to measure symptom severity and bothersomeness. The symptom diary is evaluating symptoms, bothersomeness, and impact of UTI symptoms on daily activity. Among the available PROMs measuring symptom severity and bothersomeness, the ACSS and the UTI-SIQ-8 can be recommended for use according to COSMIN criteria. Considering aspects of feasibility, an online app is available for the administration of the UTI-SIQ-8, which may encourage primary care physicians to use the UTI-SIQ-8 in their daily practice and researchers to apply it in studies involving women with uncomplicated UTIs [45]. The ACSS is a well-established instrument with easy access online to versions in 17 different languages, which likewise facilitates its use in clinical care and research. The ACSS also has important limitations. First, the ‘quality of life’ domain includes three items, of which one refers to bothersomeness of the symptoms and two to impact on work and everyday and social activities. This further emphasizes the need to analyze the measurement model. Second, the ‘differential’ domain may help clinicians in differential diagnosis from other diseases associated with dysuria, and the ACSS additionally allows for the evaluation of symptom change and treatment success. Although the ACSS appears as valuable tool for physicians, it must be considered that diagnostics and disease monitoring are not the purpose of a PROM. The ICIQ-FLUTS and the UTISA, which also aim to assess symptom severity and bothersomeness, require further validation according to COSMIN criteria. It should be noted that the ICIQ-FLUTS includes items on incontinence symptoms, and as only one content validity study of doubtful quality was available, further content validity studies in women with uncomplicated UTIs are highly recommended before applying the instrument. Concerning measures of the impact of uncomplicated UTIs on work and regular activities, we found that the AIA has the potential to be recommended, but also needs further validation before it can be used in future clinical trials. The symptom diary is the measure capturing the broadest spectrum of outcomes encompassing symptom severity, bothersomeness, and impact on daily activities. Currently, the symptom diary does not fulfill the COSMIN criteria for a recommendation, but the results of our evaluation indicate that the instrument is promising for use in medical care and research, encouraging further investigations.

When selecting a PROM, also generic instruments might be considered. In view of the treatment of uncomplicated UTIs, several alternative therapeutic options to antibiotics have been developed, and their evaluation considering the patients' perspective is indispensable. In this regard, the patient’s HRQoL is an important outcome of treatment effectiveness [50]. A previous systematic review of studies measuring the impact of UTIs on HRQoL identified two generic measures of HRQoL for use in women with symptomatic UTIs: the SF-36 and the QWB  [15]. This review, however, has not evaluated the psychometric properties of these instruments, which emphasizes the need for further research in this field. Overall, a systematic review on all available generic PROMs for use in women with uncomplicated UTIs and an evaluation of their psychometric properties would be a valuable contribution.

### Strengths and limitations

The present systematic review has several important strengths. First, we applied an established comprehensive and sensitive search filter not restricted to publication year and language. Furthermore, our search strategy included any PROMs for use in women with uncomplicated UTIs, which allows to capture all potentially relevant outcomes. Second, our literature search was carried out in the two major databases PubMed and Web of Science. We additionally searched the reference lists of the included studies for relevant articles, and contacted the authors of the included studies to obtain further information regarding known instruments and research activities in PROMs for uncomplicated UTIs. Third, the assessment of the studies was conducted according to predefined eligibility criteria and in accordance with the COSMIN guidelines. A limitation may arise from the fact that we did not search all reference lists of relevant full-texts for further eligible studies and there are further databases such as Scopus, Embase, or PsycINFO which we did not search. Since several researchers were involved in the literature screening and data extraction, the individual studies were not always rated by the same two reviewers.

## Conclusion

We identified six PROMs for use in women with uncomplicated UTIs. According to COSMIN criteria, the ACSS and the UTI-SIQ-8 can be recommended for use in future clinical trials. However, content validity is a major concern of both instruments. Although the ACSS und UTI-SIQ-8 showed sufficient content validity, the lack of patient involvement is a significant weakness indicating the need for further content validity studies. Further, the measurement model of the ACSS needs to be analyzed. A conceptual weakness of the ACSS refers to the ‘differential’ domain, which is not a PROM. Among the category B instruments, we found sufficient content validity of the symptom diary. Furthermore, patients were involved in the development, which is an important criterion [48]. The symptom diary is measuring a variety of outcomes including symptom severity, bothersomeness, and impact on daily activities, indicating that this instrument is promising for future use. Further validation studies including the assessment of structural validity are required.

## Supplementary Information

Below is the link to the electronic supplementary material.

Supplementary file1 (DOCX )

## Data Availability

The articles used and analyzed during the current study are available from the corresponding author on reasonable request.

## Code availability

All data analyses were conducted using the COSMIN manuals (available at https://www.cosmin.nl/tools/guideline-conducting-systematic-review-outcome-measures/).
