# Supplementary material for: Patient-reported outcome measures for uncomplicated urinary tract infections in women: a systematic review
Source: Qual Life Res. 2023 Feb 16;32(8):2137–53. doi: 10.1007/s11136-023-03358-5 (PMC10329060; doi:10.1007/s11136-023-03358-5)
Supplement: Supplementary file 1 — Supplementary file1 (DOCX ) [file 11136_2023_3358_MOESM1_ESM.docx]

**Appendix 1** Search strategy for PUBMED

**### A: Target population – Uncomplicated lower urinary tract infections (UTIs)**

"uncomplicated urinary tract infection"[Title/Abstract] OR "acute urinary tract infection"[Title/Abstract] OR "uncomplicated lower urinary tract infection"[Title/Abstract] OR "acute lower urinary tract infection"[Title/Abstract] OR "recurrent urinary tract infection"[Title/Abstract] OR "cystitis"[Title/Abstract] OR "uncomplicated cystitis"[Title/Abstract] OR "acute cystitis"[Title/Abstract] OR "acute uncomplicated cystitis "[Title/Abstract] OR "recurrent cystitis"[Title/Abstract] OR "recurrent acute cystitis"[Title/Abstract] OR "dysuria"[Title/Abstract] OR "suprapubic pain"[Title/Abstract] OR "haematuria"[Title/Abstract] OR "hematuria"[Title/Abstract] OR "microscopic haematuria"[Title/Abstract] OR "macroscopic haematuria"[Title/Abstract] OR "microscopic hematuria"[Title/Abstract] OR "macroscopic hematuria"[Title/Abstract] OR "pollakisuria"[Title/Abstract] OR "bacteriuria"[Title/Abstract] OR "urinary urgency"[Title/Abstract]

**### B: Construct – PROMs including Quality of Life**

("Patient Reported Outcome Measures"[Mesh] OR "Quality of Life"[Mesh] OR prom[tiab] OR proms[tiab] OR pro[tiab] OR pros[tiab] OR HRQL[tiab] OR HRQoL[tiab] OR QL[tiab] OR QoL[tiab] OR quality of life[tiab] OR life quality[tiab] OR health index*[tiab] OR health indices[tiab] OR health profile*[tiab] OR health status[tw] OR ((patient[tiab] OR self[tiab] OR carer[tiab] OR proxy[tiab]) AND ((report[tiab] OR reported[tiab] OR reporting[tiab]) OR (rated[tiab] OR rating[tiab] OR ratings[tiab]) OR based[tiab] OR (assessed[tiab] OR assessment[tiab] OR assessments[tiab]))) OR ((disability[tiab] OR function[tiab] OR functional[tiab] OR functions[tiab] OR subjective[tiab] OR utility[tiab] OR utilities[tiab] OR wellbeing[tiab] OR well being[tiab]) AND (outcome[tiab] OR outcomes[tiab] OR index[tiab] OR indices[tiab] OR instrument[tiab] OR instruments[tiab] OR measure[tiab] OR measures[tiab] OR questionnaire[tiab] OR questionnaires[tiab] OR profile[tiab] OR profiles[tiab] OR scale[tiab] OR scales[tiab] OR score[tiab] OR scores[tiab] OR status[tiab] OR survey[tiab] OR surveys[tiab])))

**### C: Measurement properties**

(instrumentation[sh] OR methods[sh] OR “Comparative Study”[pt] OR “psychometrics”[MeSH] OR psychometr*[tiab] OR clinimetr*[tw] OR clinometr*[tw] OR “outcome assessment”[tiab] OR “outcome measure*”[tw] OR “observer variation”[MeSH] OR “observer variation”[tiab] OR “Health Status Indicators”[Mesh] OR “reproducibility of results”[MeSH] OR reproducib*[tiab] OR “discriminant analysis”[MeSH] OR reliab*[tiab] OR unreliab*[tiab] OR valid*[tiab] OR “coefficient of variation”[tiab] OR coefficient[tiab] OR homogeneity[tiab] OR homogeneous[tiab] OR “internal consistency”[tiab] OR (cronbach*[tiab] AND (alpha[tiab] OR alphas[tiab])) OR (item[tiab] AND (correlation*[tiab] OR selection*[tiab] OR reduction*[tiab])) OR agreement[tw] OR precision[tw] OR imprecision[tw] OR “precise values”[tw] OR test-retest[tiab] OR (test[tiab] AND retest[tiab]) OR (reliab*[tiab] AND (test[tiab] OR retest[tiab])) OR stability[tiab] OR interrater[tiab] OR inter-rater[tiab] OR intrarater[tiab] OR intra-rater[tiab] OR intertester[tiab] OR inter-tester[tiab] OR intratester[tiab] OR intra-tester[tiab] OR interobserver[tiab] OR inter-observer[tiab] OR intraobserver[tiab] OR intra-observer[tiab] OR intertechnician[tiab] OR inter-technician[tiab] OR intratechnician[tiab] OR intra-technician[tiab] OR interexaminer[tiab] OR inter-examiner[tiab] OR intraexaminer[tiab] OR intra-examiner[tiab] OR interassay[tiab] OR inter-assay[tiab] OR intraassay[tiab] OR intra-assay[tiab] OR interindividual[tiab] OR inter-individual[tiab] OR intraindividual[tiab] OR intra-individual[tiab] OR interparticipant[tiab] OR inter-participant[tiab] OR intraparticipant[tiab] OR intra-participant[tiab] OR kappa[tiab] OR kappa’s[tiab] OR kappas[tiab] OR repeatab*[tw] OR ((replicab*[tw] OR repeated[tw]) AND (measure[tw] OR measures[tw] OR findings[tw] OR result[tw] OR results[tw] OR test[tw] OR tests[tw])) OR generaliza*[tiab] OR generalisa*[tiab] OR concordance[tiab] OR (intraclass[tiab] AND correlation*[tiab]) OR discriminative[tiab] OR “known group”[tiab] OR “factor analysis”[tiab] OR “factor analyses”[tiab] OR “factor structure”[tiab] OR “factor structures”[tiab] OR dimension*[tiab] OR subscale*[tiab] OR (multitrait[tiab] AND scaling[tiab] AND (analysis[tiab] OR analyses[tiab])) OR “item discriminant”[tiab] OR “interscale correlation*”[tiab] OR error[tiab] OR errors[tiab] OR “individual variability”[tiab] OR “interval variability”[tiab] OR “rate variability”[tiab] OR (variability[tiab] AND (analysis[tiab] OR values[tiab])) OR (uncertainty[tiab] AND (measurement[tiab] OR measuring[tiab])) OR “standard error of measurement”[tiab] OR sensitiv*[tiab] OR responsive*[tiab] OR (limit[tiab] AND detection[tiab]) OR “minimal detectable concentration”[tiab] OR interpretab*[tiab] OR ((minimal[tiab] OR minimally[tiab] OR clinical[tiab] OR clinically[tiab]) AND (important[tiab] OR significant[tiab] OR detectable[tiab]) AND (change[tiab] OR difference[tiab])) OR (small*[tiab] AND (real[tiab] OR detectable[tiab]) AND (change[tiab] OR difference[tiab])) OR “meaningful change”[tiab] OR “ceiling effect”[tiab] OR “floor effect”[tiab] OR “Item response model”[tiab] OR IRT[tiab] OR Rasch[tiab] OR “Differential item functioning”[tiab] OR DIF[tiab] OR “computer adaptive testing”[tiab] OR “item bank”[tiab] OR “cross-cultural equivalence”[tiab])

**### D: Feasibility of PROMs**

(((accepta*[Title/Abstract]) OR ("ease of use"[Title/Abstract])) OR (practica*[Title/Abstract])) OR (feasib*[Title/Abstract])

**### E: Individual UTI-specific PROMs**

("Acute Cystitis Symptom Score"[Title/Abstract] OR "Activity Impairment Assessment"[Title/Abstract] OR "UTI Symptom Assessment questionnaire"[Title/Abstract] OR ("urinary tract infection"[Title/Abstract] AND "diary"[Title/Abstract]))

**### F: Exclusion filter**

("addresses" OR "biography" OR "case reports" OR "comment" OR "directory" OR "editorial" OR "festschrift" OR "interview" OR "lectures" OR "legal cases" OR "legislation" OR "letter" OR "news" OR "newspaper article" OR "patient education handout" OR "popular works" OR "congresses" OR "consensus development conference" OR "consensus development conference, nih" OR "practice guideline" OR (exp "animals"/ NOT "humans”))

**Appendix 2** Questionnaires of the identified patient-reported outcome measures

**Acute Cystitis Symptom Score (ACSS)**

All translated and validated versions of the ACSS are available online: http://www.acss.world/downloads.html

**Activity Impairment Assessment (AIA)**

The English version of the AIA is presented in the research paper: https://www.ncbi.nlm.nih.gov/pmc/articles/PMC1180845/

**International Consultation on Incontinence Questionnaire Female Lower Urinary Tract Symptoms (ICIQ-FLUTS)**

The English version of the ICIQ-FLUTS is available online: https://iciq.net/iciq-fluts

**Symptom diary**

The symptom diary is not publicly available.

**Urinary Tract Infection Symptom Assessment Questionnaire (UTISA)**

The English version of the UTISA is presented in the research paper: https://bjui-journals.onlinelibrary.wiley.com/doi/10.1111/j.1464-410X.2005.05630.x

**Urinary tract infection-Symptom and Impairment Questionnaire (UTI-SIQ-8)**

The German and English version of the UTI-SIQ-8 is available online as appendix of the research paper: https://www.ncbi.nlm.nih.gov/pmc/articles/PMC7887375/#SP1

**Appendix 3** COSMIN Risk of Bias overall ratings for content validity studies

|  | **ACSS** | **AIA** | **ICIQ-FLUTS** | **Symptom diary** | **UTISA** | **UTI-SIQ-8** |
| --- | --- | --- | --- | --- | --- | --- |
| **Box 1. PROM development** | Doubtful^a^ | Inadequate^c^ | Inadequate^e^ | Doubtful^g^ | Inadequate^h^ | Inadequate^i^ |
| **Box 2. Content validity** | Doubtful^b^ | Doubtful^d^ | Doubtful^f^ | Doubtful^g^ | **-** | **-** |

*COSMIN* Consensus-based Standards for the selection of health Measurement Instruments, *PROM* patient-reported outcome measure

*ACSS* Acute Cystitis Symptom Score, *AIA* Activity Impairment Assessment Score, *ICIQ-FLUTS* International Consultation on Incontinence Questionnaire Female Lower Urinary Tract Symptoms, *UTISA* Urinary Tract Infection Symptom Assessment Questionnaire, *UTI-SIQ-8* Urinary Tract Infection – Symptom and Impairment Questionnaire

^a^Alidjanov et al. 2014a

^b^Alidjanov et al. 2014b, 2015, 2017; Bruyère et al. 2022; Choi et al. 2022; Magyar et al. 2018; Stamatiou et al. 2021b

^c^Wild et al. 2005

^d^Vincent et al. 2020

^e^Jackson et al. 1996

^f^Chattrakulchai et al. 2020

^g^Holm et al. 2017

^h^Colgan et al. 1994

^i^Gágyor et al. 2021

**Appendix 4** Content validity rating of the included PROMs

|  |  | **Relevance** | **Comprehensiveness** | **Comprehensibility** | **Content validity rating** |
| --- | --- | --- | --- | --- | --- |
| ACSS | Overall rating | + | + | + | Sufficient (+) |
|  | Quality of evidence | Moderate (due to risk of bias) | | | |
|  |  |  |  |  |  |
| AIA | Overall rating | + | - | ± | Inconsistent (±) |
|  | Quality of evidence | No grading if overall rating is inconsistent | | | |
|  |  |  |  |  |  |
| ICIQ-FLUTS | Overall rating | - | - | + | Inconsistent (±) |
|  | Quality of evidence | No grading if overall rating is inconsistent | | | |
|  |  |  |  |  |  |
| Symptom diary | Overall rating | + | + | + | Sufficient (+) |
|  | Quality of evidence | Moderate (due to risk of bias) | | | |
|  |  |  |  |  |  |
| UTISA | Overall rating | + | ± | + | Sufficient (+) |
|  | Quality of evidence | Very low (due to risk of bias) | | | |
|  |  |  |  |  |  |
| UTI-SIQ-8 | Overall rating | + | + | + | Sufficient (+) |
|  | Quality of evidence | Very low (due to risk of bias) | | | |

*ACSS* Acute Cystitis Symptom Score, *AIA* Activity Impairment Assessment Score, *ICIQ-FLUTS* International Consultation on Incontinence Questionnaire Female Lower Urinary Tract Symptoms, *UTISA* Urinary Tract Infection Symptom Assessment Questionnaire, *UTI-SIQ-8* Urinary Tract Infection – Symptom and Impairment Questionnaire
